# Interaction of surface glycoprotein of SARS-CoV-2 variants of concern with potential drug candidates: A molecular docking study

**DOI:** 10.12688/f1000research.109586.1

**Published:** 2022-04-07

**Authors:** Anuj Mavlankar, Afzal Ansari, Mukul Sharma, Purna Dwivedi, Pushpendra Singh

**Affiliations:** 1 Microbial Pathogenesis and Genomics Laboratory, ICMR National Institute of Research in Tribal Health, Jabalpur, Madhya Pradesh, 482003, India; 2 Maharaja Sayajirao University of Baroda, Vadodara, Gujarat, 390002, India

**Keywords:** Molecular docking, SARS-CoV-2, VOCs, Drug repurposing

## Abstract

**Background:** COVID-19 has become a global threat. Since its first outbreak from Wuhan, China in December 2019, the SARS-CoV-2 virus has gone through structural changes arising due to mutations in its surface glycoprotein. These mutations have led to the emergence of different genetic variants threatening public health due to increased transmission and virulence. As new drug development is a long process, repurposing existing antiviral drugs with potential activity against SARS-CoV-2 might be a possible solution to mitigate the current situation.

**Methods:** This study focused on utilizing molecular docking to determine the effect of potential drugs on several variants of concern (VOCs). The effect of various drugs such as baricitinib, favipiravir, lopinavir, remdesivir and dexamethasone, which might have the potential to treat SARS-CoV-2 infections as evident from previous studies, was investigated for different VOCs.

**Results:** Remdesivir showed promising results for B.1.351 variant (binding energy: -7.3 kcal/mol) with residues Gln319 and Val503 facilitating strong binding. Favipiravir showed favorable results against B.1.1.7 (binding energy: -5.6 kcal/mol), B.1.351 (binding energy: -5.1 kcal/mol) and B.1.617.2 (binding energy: -5 kcal/mol). Molecular dynamics simulation for favipiravir/B.1.1.7 was conducted and showed significant results in agreement with our findings.

**Conclusions:** From structural modeling and molecular docking experiments, it is evident that mutations outside the receptor binding domain of surface glycoprotein do not have a sharp impact on drug binding affinity. Thus, the potential use of these drugs should be explored further for their antiviral effect against SARS-CoV-2 VOCs.

## Abbreviations

COVID-19: Coronavirus disease 2019

FDA: Food and Drug Administration

GISAID: Global initiative on sharing all influenza data

hACE2: Human angiotensin converting enzyme

MD: Molecular dynamics

RBD: Receptor binding domain

RMSD: Root mean square deviation

SARS-CoV-2: Severe Acute Respiratory Syndrome Coronavirus-2

VOCs: Variant of Concern

## Introduction

The spread of human coronavirus SARS-CoV-2 has been increasing since it was first detected in the Chinese city of Wuhan in December 2019. ^1^ Several efforts have been taken to prevent its spread after the World Health Organization (WHO) declared it a public health emergency on January 31, 2020. However, its continual spread across the world compelled the WHO to declare it a pandemic. ^2^ Different genetic variants of this novel coronavirus have appeared and been transmitted across the world amidst the pandemic. ^3^ WHO classified some of these genetic variants into three categories: variant of interest (VOI), variant of concern (VOC) and variants of high consequence (VOHC). ^4^ Five different genetic variants have been placed in the VOC category, due to their increased transmission rates, more severe disease, or significant reduction in antibodies generated due to previous infection or vaccination namely by B.1.1.7 (Alpha), B.1.351 (Beta), B.1.617.2 (Delta), AY.1 (Delta plus) and P.1 (Gamma). Certain anti-retroviral drugs have been used owing to their promising results for the emergency treatment of COVID-19 patients. ^5^ Remdesivir was the first drug to be approved by the United States Food and Drug Administration (USFDA) for the treatment of COVID-19 patients. ^6^ However, emerging mutations in drug targets (such as the receptor binding domain [RBD] of the surface glycoprotein) are likely to affect the binding affinity through altered drug-receptor interaction. ^7^ Considering the emergence of several VOCs in different parts of the world, it is important to ascertain the effect of their signature genomic variants such as single nucleotide polymorphisms (SNPs) and insertions/deletions (InDels). To the best of our knowledge, there is a paucity of such information, especially regarding the newly emerged Delta variant. ^8^ Docking studies are very helpful and serve as the first starting point for such investigations. ^9^ Therefore, in this study, surface glycoprotein sequences of different VOCs were modeled *in silico* and their interactions with the drugs baricitinib, dexamethasone, favipiravir, lopinavir and remdesivir were studied using molecular docking. These drugs have shown promising results in various clinical studies and thus have been considered to determine their binding affinity on SARS-CoV-2 VOCs. ^10^^–^^12^ The main aim of this study was to utilize an *in silico* docking approach to estimate relative changes in the binding affinity of these potential drugs against the characteristic mutational profile of the spike protein sequence in different VOCs, to predict their potential therapeutic efficacy against VOCs.

### Spike mutations

The surface glycoprotein (Spike) allows the virus to bind to hACE2 receptors and thereby promotes the virus’s entry into the host cell. ^13^^,^^14^ It is divided into two subunits, S1 and S2. The S1 subunit consists of the RBD which directly binds to hACE2. It is also the target of neutralizing antibodies. Thus, it is the region with most mutations with clinical significance in terms of viral transmissibility and virulence. ^15^^,^^16^ Major mutations reported in different VOCs are shown in Table 1.

**Table 1.** Spike mutations reported in variants of concern VOCs. Mutations in bold represent its presence in different variants.

Variant of concern (VOCs)				
B.1.1.7 (Alpha)	B.1.351 (Beta)	P.1 (Gamma)	B.1.617.2 (Delta)	AY.1 (Delta plus *)
H69del/V70del/Y144del	D80A	L18F	T19R	**K417N**
**N501Y**	D215G	T20N	G142D	**L452R**
A570D	L242del/A243del/L244del	P26S	E156G	**T478K**
**D614G**	**K417N**	D138Y	F157del/R158del	**D614G**
P681H	**E484K**	R190S	**L452R**	**P681R**
T716I	**N501Y**	K417T	**T478K**	
S982A	**D614G**	**E484K**	**D614G**	
D1118H	A701V	**N501Y**	**P681R**	
		**D614G**	D950N	
		H655Y		
		T1027I		
		V1176F		

* AY.1 is commonly referred as Delta plus, ^17^ although this is not as per WHO classification which considers it as one of the types within Delta lineage.

## Methods

### Dataset collection and mutation analysis

A total of 24 full-length sequences of SARS-CoV-2 genomes categorized into five VOCs from different geographical regions were selected and retrieved from the Global Initiative on Sharing All Influenza Data ( GISAID) database. ^18^^,^^19^ The first sequence of SARS-CoV-2 originating from Wuhan was retrieved from the National Center for Biotechnology Information ( NCBI) nucleotide database as a reference (NCBI reference sequence: NC_045512.2). Mutation analysis was carried out by multiple sequence alignment of the retrieved sequences using the ClustalW algorithm in MEGA-X software v 10.2.3 ^20^ and mutation positions were determined. This analysis was performed to check the frequency of mutations across sequences from different VOCs. After this, a particular mutation was inserted in the sequence of a VOC, followed by its modeling. As these sequences were derived from COVID-19 infected patients, they represent the actual frequency of genetic mutations acquired by SARS-CoV-2. It reveals that once a specific mutation in any variant has evolved, it remains conserved in the descendant population, which may again acquire new characteristic mutations.

### 3D structure prediction, model quality assessment and validation

The novel SARS-CoV-2 surface glycoprotein nucleotide wild type (WT) gene sequence NC_045512.2 was retrieved from the NCBI ^21^ nucleotide database. Reported mutations were induced in the retrieved sequence. A homology model was built for the surface glycoprotein of SARS-CoV-2 VOCs using SWISS-MODEL software. ^22^ The matched templates were Protein Data Bank (PDB) ID 7N1U, chain A for B.1.1.7; PDB ID 7N1Q, chain A for B.1.315; PDB ID 7KRS, chain A for B.1.617.2, AY.1 and P.1. The Duke University MolProbity web server ^23^^,^^24^ and the University of California Structure Analysis and Verification Server ( SAVES) ^25^ were used to examine the modeled structure. Several other online tools such as PROCHECK, ^26^^,^^27^ Verify3D ^28^^,^^29^ and ERRAT ^25^^,^^30^ were further used to check the validity of the predicted models. Minimization of the model was carried out after addition of missing hydrogens to prepare it for molecular docking. ^31^

### Molecular docking

AutoDock Vina 1.1.2 software was used for molecular docking experiments. ^32^^,^^33^ The modeled surface glycoprotein of all SARS-CoV-2 VOCs was served as binding target and five approved drugs as ligand. All the compounds were first optimized in their active forms in physiological conditions.

### Protein and ligand preparation

The structure of investigated drugs, namely baricitinib (PubChem CID 44205240), favipiravir (PubChem CID 492405), lopinavir (PubChem CID 92727), remdesivir (PubChem CID 121304016) and dexamethasone (PubChem CID 5743) were retrieved from PubChem database. ^34^ AutoDockTools 1.5.6, a free graphical user interface of MGL software package was used for all the required file conversions needed for the docking study. ^35^^,^^36^ The rotatable bonds present on the ligands were treated as non-rotatable for performing the docking. All the water molecules and hetero atoms present on the receptor surface were removed, followed by the addition of Kollman charges and polar hydrogen atoms using AutoDockTools 1.5.6. The Gasteiger charge calculation method and partial charges were also applied to the ligand molecules. ^37^

### Grid box preparation and docking

Molecular Docking was performed with modeled surface glycoproteins of different VOCs as receptors and selected drugs as ligands. Grid box parameters were selected using AutoDockTools 1.5.6 ( Table 2). The Lamarckian Genetic Algorithm was used for performing docking to explore the conformational space required for the ligand with a population size of 150 individuals. The total number of current grid points per map was 64,000. Other parameters were set at default.

**Table 2.** Grid box parameters selected for surface glycoprotein of different variants of concern (VOCs).

Variant of concern (VOC)	Center grid box (points)	Number of points (x, y, z)	Spacing (Å)
Wild type sequence of reference strain	173.286 × 144.433 × 154.486	40 × 40 × 40	0.375
B.1.1.7 (Alpha)	215.849 × 187.327 × 197.160	40 × 40 × 40	0.375
B.1.351 (Beta)	199.651 × 220.357 × 196.477	40 × 40 × 40	0.375
B.1.617.2 (Delta)	217.083 × 242.632 × 219.461	40 × 40 × 40	0.375
AY.1 (Delta plus)	216.067 × 187.052 × 197.514	40 × 40 × 40	0.375
P.1 (Gamma)	216.204 × 187.050 × 197.452	40 × 40 × 40	0.375

### Molecular dynamics (MD) simulation

To check the validity of molecular docking results for favipiravir against the B.1.1.7 (Alpha) variant, a molecular dynamics simulation was conducted. The simulation was conducted in GROMACS 2018, ^38^^,^^39^ with CHARMM36 as all-atom force field. ^40^ For the ligand-receptor complex, all receptors missing hydrogen atoms were added using Chimera. ^41^^,^^42^ The protein-ligand complex was placed in an isotonic box with a dodecahedron cell. The box contained a neutralizing number of sodium (Na ^+^) and chloride (Cl ^-^) ions based on the total charge of the protein. Topology parameters for the ligand were built using the CHARMM General Force Field (CGenFF) tool ^43^ to generate the CHARMM36 parameters. The solvation step was followed by energy minimization, equilibration number of particles volume temperature (NVT) ensemble and number of particles pressure temperature (NPT) ensemble; then, MD simulation with 2 femtoseconds (fs) integration steps for 20 ns were conducted. The output trajectory was then subjected to Periodic Boundary Conditions (PBC) correction and the system was fitted to its start position based on the backbone of the receptor. Further analysis was performed to plot the root-mean-square deviation (RMSD) of the ligand and Molecular Mechanics Poisson-Boltzmann Surface Area (MMPBSA) energy computation.

## Results and discussion

### Multiple sequence alignment and structure analysis

The mutation analysis was carried out for the surface glycoprotein of SARS-CoV-2 VOC, and random clinical samples from different geographical regions were retrieved from the GISAID database ^18^ to verify the known mutations present in variants and to reveal any other significant mutation present, if any. Multiple sequence alignment of different clinical samples with the surface glycoprotein of B.1.1.7 revealed characteristic mutations such as Del 69/70, Del 144/145, N501Y, A570D, D614G, P681H, T716I, S982A, and D1118H. Similar steps were performed for another SARS-CoV-2 VOC (Supplementary Figure 1, *Extended data* ^44^). However, no novel mutation was reported. The reported mutations which had a frequency of occurrence of 50% and above were considered for surface glycoprotein modeling for VOCs ( Table 1). A high-quality model was constructed for the surface glycoprotein of SARS-CoV-2 based on the matching templates (7KRS, 7N1U and 7N1Q) for different SARS-CoV-2 VOCs. The 7KRS PDB accession number is a viral protein complex characterized by the mutation D614G solved by electron microscopy with a resolution of 3.20 Å. ^45^ It has a sequence similarity of 99.14% with the B.1.617.2, AY.1 and P.1 variants. 7N1U and 7N1Q are the PDB accession numbers of viral protein structures solved by electron microscopy, with a resolution of 3.1 Å and 2.90 Å respectively. ^46^ These were the matching templates for B.1.1.7 and B.1.351 with 99.80% and 99.92% sequence similarity respectively.

### Binding interactions of drugs with the SARS-CoV-2 surface glycoprotein

The docking results are shown in terms of binding energy ( Table 3) and number of interacting amino acid residues (Supplementary Table 1, *Extended data* ^44^) at the active site of SARS-CoV-2 surface glycoprotein. The binding affinity of each drug for different variants was computed by assuming 100% binding affinity with SARS-CoV-2 WT. The lowest binding energy and RMSD conformation was considered as the most suitable docking pose. The binding interactions between different drugs and surface glycoprotein of VOCs were prepared, visualized and analyzed using PyMOL v 2.5.2 and Discovery Studio 2021. ^47^^–^^49^

**Table 3.** Resultant binding energy (kcal/mol) after molecular docking of SARS-CoV-2 surface glycoprotein of Variant of Concern (VOCs) against different drugs.

Variant/drug	Baricitinib		Favipiravir		Lopinavir		Remdesivir		Dexamethasone	
	Binding energy (Kcal/mol)	Affinity (%)	Binding energy (Kcal/mol)	Affinity (%)	Binding energy (Kcal/mol)	Affinity (%)	Binding energy (Kcal/mol)	Affinity (%)	Binding energy (Kcal/mol)	Affinity (%)
SARS-CoV-2 wild type	-6.7	100	-4.9	100	-10.1	100	-7	100	-8	100
SARS-CoV-2 B.1.617.2 (Delta)	-6.3	94.02	-5	102.05	-9.2	91.08	-6.7	95.71	-7.7	96.25
SARS-CoV-2 AY.1 (Delta plus)	-6.4	95.52	-4.8	97.95	-8.7	86.13	-6.7	95.71	-4.4	55
SARS-CoV-2 B.1.1.7 (Alpha)	-6.6	98.50	-5.6	114.28	-8.8	87.12	-7	100	-5.7	71.25
SARS-CoV-2 B.1.351 (Beta)	-7.1	105.97	-5.1	104.08	-10.1	100	-7.3	104.28	-5.8	72.50
SARS-CoV-2 P.1 (Gamma)	-6.4	95.52	-4.9	100	-8.8	87.12	-6.3	90	-6.2	77.50

**Figure 1.**
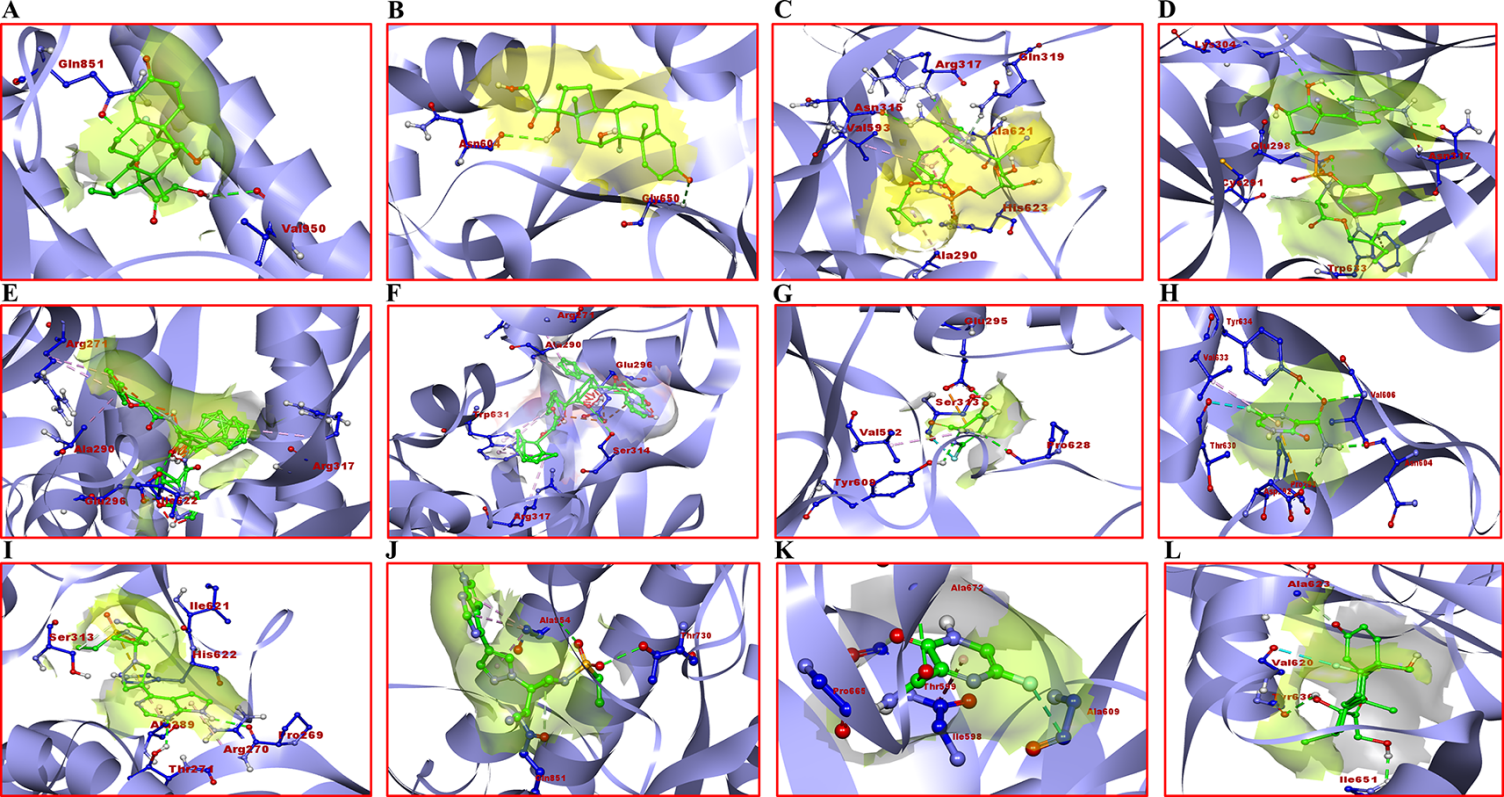
Binding interactions of SARS-CoV-2 surface glycoprotein with selected drugs of high and low binding energies for different variants of concern (VOCs). A) B.1.617.2 with dexamethasone; B) AY.1 with dexamethasone; C) B.1.351 with remdesivir; D) P.1 with remdesivir; E) B.1.351 with lopinavir; F) AY.1 with lopinavir; G) B.1.1.7 with favipiravir; H) AY.1 favipiravir; I) B.1.351 with baricitinib; J) B.1.617.2 with baricitinib; K) wild type; (WT) with favipiravir; L) WT with dexamethasone.

Dexamethasone showed a sharp difference between binding energies with the SARS-CoV-2 surface glycoprotein of B.1.617.2 (the Delta VOC, binding energy of -7.7 Kcal/mol) compared to that of AY.1 (the Delta plus VOC, with a binding energy of -4.4 Kcal/mol). It formed two H-bonds with Gln851 and Val950 amino acid residues in the active site region of the surface glycoprotein of B.1.617.2. In addition, it formed two H-bonds with Asn604 and Gly650 with the surface glycoprotein of AY.1. It showed 96.25% and 55% binding affinity with B.1.617.2 and AY.1 respectively, compared to WT (reference, 100%). In contrast, favipiravir showed the highest binding affinities for all variants except AY.1 in comparison to WT. It showed a maximum binding affinity of 114.28% (binding energy: -5.6 Kcal/mol) and formed conventional H-bonds with Glu295, Tyr609 and Pro628 amino acid residues in the active pocket region of surface glycoprotein of B.1.1.7 (Alpha). Remdesivir appeared to be quite effective in binding the active pocket region of different SARS-CoV-2 variants’ surface glycoproteins. It showed a highest binding affinity of 104.28% (binding energy: -7.3 Kcal/mol) with B.1.351 (beta) as compared to WT. It formed three conventional H-bonds with amino acid residues Asn315, Arg317 and His617, one carbon-hydrogen bond with Gln319, and one alkyl bond with Ala290, Val503 and Ala621. Two amino acid residues, i.e., Gln319 and Val503 are present in the RBD of SARS-CoV-2 which facilitate its binding to the hACE2. ^50^ Similarly, baricitinib showed a strong binding affinity of 105.97% (binding energy: -7.1 Kcal/mol) with B.1.351 and formed H-bond with Pro269 and Thr271 when compared to WT. Lopinavir also showed significant results with a binding affinity of 100% (binding energy: -10.1 Kcal/mol) with the surface glycoprotein of B.1.351 as compared to WT and other variants. It showed the formation of an H-bond with Ile622. The selected 3D structural view of SARS-CoV-2 surface glycoprotein docking with different drugs and amino acid binding residues is shown in Figure 1 and Supplementary Table 1 ( *Extended data* ^44^) respectively. Receptor amino acid residues of proteins are shown in blue and ligands are presented in green. Other docking images are shown in Supplementary Figure 3 ( *Extended data* ^44^).

The emergence of new SARS-CoV-2 variants leads to the need for new treatment drugs development, which is a long process. However, with an increased patient mortality ratio, it is of utmost importance to repurpose existing drugs used to treat other viral diseases. ^51^ Failure to target the gene encoding the surface glycoprotein has been observed as SARS-CoV-2 variants are detected. ^7^ According to the latest guidelines of the Indian Council of Medical Research, approved test kits must employ multiplex RT-PCR assays, as the tests assessing only the surface glycoprotein may fail and produce false negative results.

The viral entry into the host cell is facilitated by its successful binding to the angiotensin converting enzyme (ACE2) receptor. ^52^ Overexpression of ACE2 may lead to disease severity as observed in mice. ^53^ Lung damage can be reversed by blocking the renin-angiotensin pathway. ^54^ A recent study had shown that the surface glycoprotein of SARS-CoV-2 binds to ACE2 with a 10- to 20- fold higher affinity than other SARS-CoV surface glycoproteins, ^55^ which might be the reason for the high infectivity of SARS-CoV-2. Thus, viral entry into the host cell is a vital step which must be exploited for an efficient therapeutic development. There is a rapid ongoing search for therapeutic agents against SARS-CoV-2. Various computational studies have been conducted to discover potential drugs against SARS-CoV-2. ^51^^,^^56^^,^^57^ Recent studies have been based on the drugs targeting either surface glycoprotein or main protease of SARS-CoV-2. These approaches have led to the discovery of molecules with high binding affinities to these proteins. ^58^

The molecular docking analysis of surface glycoproteins with selected drugs for different VOCs along (Supplementary Figure 2, *Extended data* ^44^) revealed promising results for B.1.351 (Beta variant). Three drugs, namely baricitinib, lopinavir and remdesivir, showed maximum binding affinities against the Beta variant as compared to the WT and other VOCs. Other variants also expressed significant binding energies. As per a recent study, ^10^ the combination of baricitinib and remdesivir was more effective than Remdesivir alone and thus helped to lower the recovery time and accelerate the clinical status of patients suffering from COVID-19, especially those requiring high-inflow oxygen ventilation. Remdesivir efficiently inhibits the replication of SARS-CoV-2 by causing delayed chain termination when getting incorporated into the viral RNA. ^59^ However, it also showed considerable binding affinity when docked with the surface glycoprotein. ^60^ Here, our molecular docking study revealed its potential as an effective drug against SARS-CoV-2 VOCs. The high energy score resulting from these docking analyses suggests that these drugs may be recommended for administration to patients with B.1.351 infection. Molecular docking revealed that two RBD residues, namely Gln319 and Val503 facilitated a strong binding. Upon comparison with the WT, favipiravir showed a significant binding affinity with B.1.1.7, B.1.617.2 and B.1.351. Favipiravir is a purine analog which inhibits the elongation phase of RNA synthesis. Favipiravir was proven to be effective in viral clearance and fast clinical improvement. ^61^ It has shown positive results in COVID-19 patients by improving patient’s health. ^62^ In concordance with our findings, favipiravir was successfully docked with the surface glycoprotein of B.1.1.7 (Alpha variant). Dexamethasone has been widely used as a therapeutic intervention to treat COVID-19 patients. The docking score of dexamethasone with the surface glycoprotein of B.1.617.2 (Delta variant) was the highest (binding energy: -7.7 Kcal/mol) compared to other variants of concern. In contrast, the docking score for AY.1 (Delta plus variant) showed the lowest affinity with dexamethasone (binding energy: -4.4 Kcal/mol). This observation shows that dexamethasone binding to the surface glycoprotein of the SARS-CoV-2 Delta variant (which has spread as one of the most dominant lineage worldwide) may represent an additional contribution to its efficacy in treating COVID-19. ^63^ Lopinavir is a drug approved by the FDA and serves as a protease inhibitor commonly used in the treatment of the Human Immunodeficiency Virus (HIV); it may be considered useful in the treatment against SARS-CoV-2 infection. ^64^ Our findings reveal that it may be a suitable choice for treatment as it shows significant binding with different VOCs, especially with B.1.351 (Beta variant; binding energy: -10.1 Kcal/mol). Remdesivir has been found to be a more potent drug than lopinavir, both *in vitro* and in MERS-CoV infected mice. ^65^ In concordance with our findings, remdesivir has shown more significant binding with B.1.351 than lopinavir as compared to WT. There is evidence of lopinavir being selective against other coronaviruses. ^12^ Despite the high binding energy with surface glycoproteins, our results encourage further *in vitro* and *in vivo* investigations. In comparison to the WT, the binding residues for different VOCs vary and some of them lie outside the receptor binding domain of the surface glycoprotein which does not have a direct role in drug affinity. However, they may impact the interaction of drug with surface glycoprotein through weak molecular interactions.

### MD simulation and RMSD

**Figure 2.**
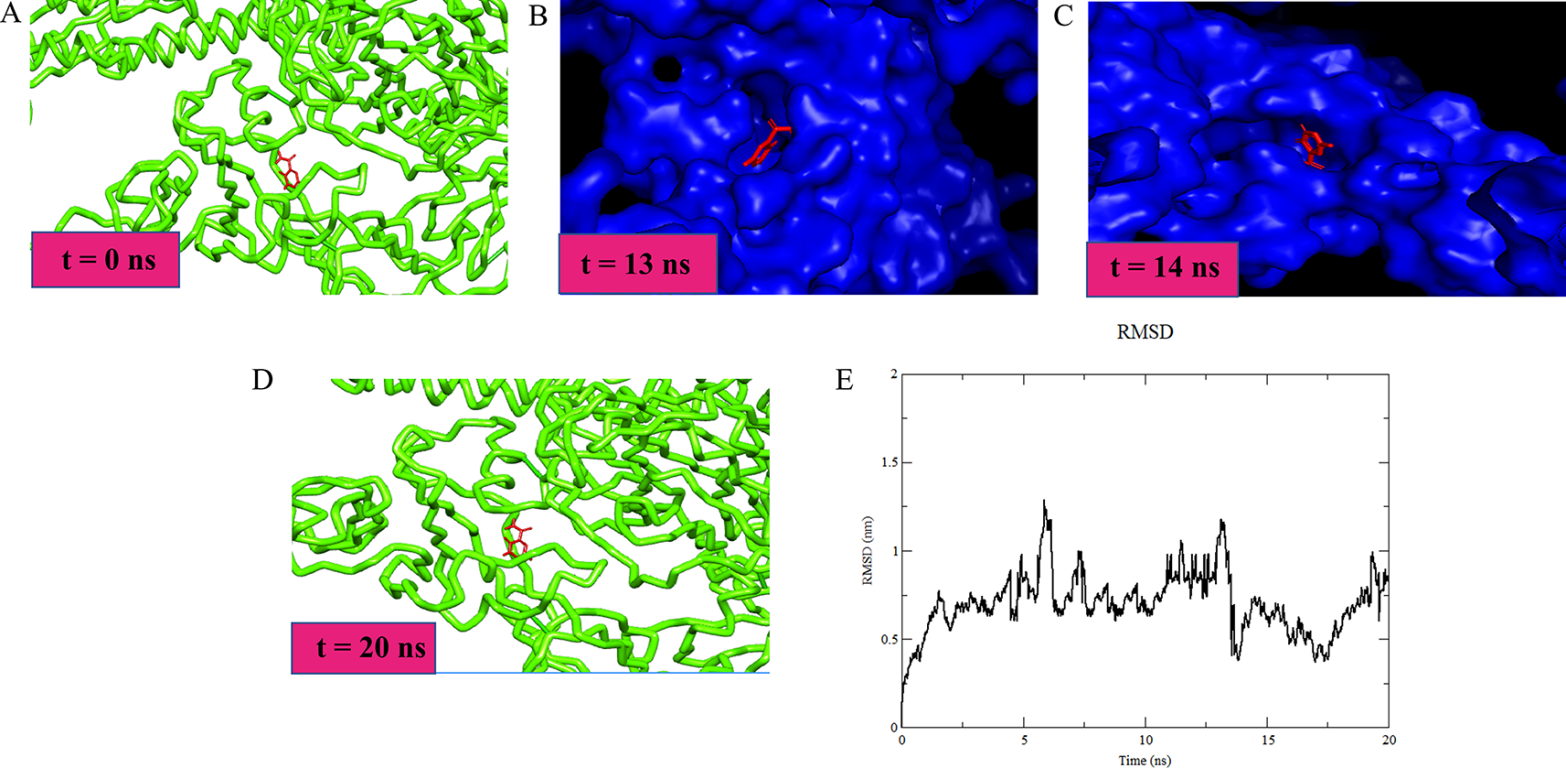
Favipiravir-Alpha variant complex molecular dynamics simulation: (A) Favipiravir/Spike complex (t = 0 ns) (B) Favipiravir inside the binding pocket of the spike protein (t = 13 ns) (C) Favipiravir/Spike, the pocket was obliterated, however, favipiravir was still attached to the spike (t = 14 ns) (D) At the end of simulation, favipiravir was in place at its binding pocket (t = 20ns) (E) Root mean square deviation plot for the favipiravir/Spike complex.

The studied favipiravir-Alpha variant (B.1.1.7) ligand heavy atoms complex showed a RMSD of 1.028 Å. The Lennard-Jones potential and the binding potential of the complex was calculated to be -129.178 kJ/mol and -137.227 kJ/mol respectively. During the initial simulation run, up to 4 ns, the ligand-receptor showed a high RMSD value, which may be due to the stearic changes the protein underwent. The ligand managed to follow the change, keeping a stable number of H-bonds. The ligand initially stabilized at the binding pocket with some additional hydrophobic interactions. At t =14 ns, the pocket was obliterated, however, the ligand was kept near to the pocket until the pocket was opened. Subsequently, the ligand was restored to its original position ( Figure 2). MD simulation for favipiravir/Alpha variant was performed as it showed a significant binding affinity compared to other drugs under study. A similar procedure can be incorporated to conduct the simulation studies on other variants with drugs showing significant binding.

## Conclusions

Drug repurposing may help to discover and identify the potential therapeutic effect of existing drugs against the genomic targets of SARS-CoV-2 virus. This study shows that the mutations (except Gln319 and Val503) outside the RBD of the surface glycoprotein of several VOCs do not largely affect the binding affinity of these drugs. No drastic structural change has been observed in variants irrespective of binding with the residues occurring outside the RBD of the surface glycoprotein. However, favipiravir showed the highest binding affinity against the Alpha variant, whereas dexamethasone showed approximately a 50% reduction in its binding affinity with the Delta plus variant when compared to the Delta variant, revealing that dexamethasone bound to surface glycoprotein of the Delta variant more strongly than the Delta plus variant. These residual fluctuations may play a role in antibody evasion and their molecular roles should be explored further. ^66^ However, the candidate drugs besides favipiravir and dexamethasone showed no significant alteration in the surface glycoprotein structure when compared to WT, implying that the current regimen of approved drugs can be continued in patients infected with these SARS-CoV-2 strains.

**Figure 3.**
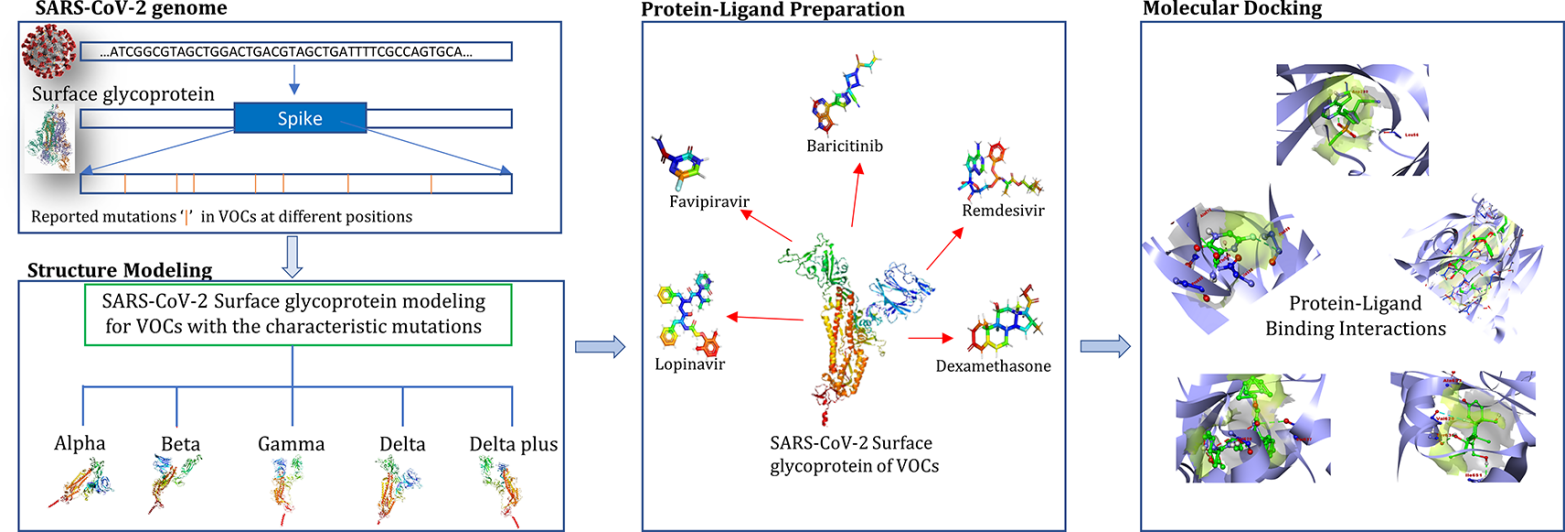
Summarized representation of the study.

Further, molecular docking approaches offer great promise for predicting, shortlisting and quickly evaluating the anti-SARS-CoV-2 potential of candidate and existing drugs which can help timely effective interventions. The workflow depicting the study has been summarized in Figure 3.

## Data availability

### Underlying data

Zenodo: Interaction of Surface Glycoprotein of SARS-CoV-2 variants of concern with Potential Drug Candidates: A Molecular Docking Study, https://doi.org/10.5281/zenodo.6339952. ^44^

This project contains the following underlying data:

- Drugs.zip (tested drug pdb files)
- Protein-structure-3D.zip
- sequences_for_mutation_frequency_analysis.xlsx
- spike_protein_sequences.docx

### Extended data

Zenodo: Interaction of Surface Glycoprotein of SARS-CoV-2 Variants of concern with Potential Drug Candidates: A Molecular Docking Study, https://doi.org/10.5281/zenodo.6339952. ^44^

This project contains the following extended data:

- Supplementary Figure 1.docx
- Supplementary Figure 2.docx
- Supplementary Figure 3.docx
- Supplementary Table 1.csv

Data are available under the terms of the Creative Commons Attribution 4.0 International license (CCBY 4.0).
