# Supplementary material for: Interaction of surface glycoprotein of SARS-CoV-2 variants of concern with potential drug candidates: A molecular docking study
Source: F1000Res. 2022 Apr 7;11:Chem Inf Sci-400. [Version 1] doi: 10.12688/f1000research.109586.1 (PMC9445560; doi:10.12688/f1000research.109586.1)
Supplement: Peer review history

# Peer review: Reviewer response for version 1

**DOI:** 10.5256/f1000research.121103.r148617

**Published:** 2022-09-27

**Authors:** Thomas Caulfield

**Affiliations:** 1 Mayo Clinic, Jacksonville, USA

NEEDS MAJOR REVISIONS.

 I like the aim and ideas. But not a very good realization of their plans.

1. Time of MD 20 ns simulations is entirely too short. And for gromacs simulations, this is not sufficient sampling time for relaxation time of any protein. It should be more like 60-100 ns for relaxation and then 100-500 ns of production run (minimum). They did not provide RMSD for Spike protein in free state (we can see that). Fig2 shows relaxation period is absent…
2. Then they try to compare free energy of docking and primitive "The LennardJones potential and the binding potential of the complex " -- they can continue Molecular dynamics stimulation and recalculate energy by g_mmpbsa https://rashmikumari.github.io/g_mmpbsa/ to get similar dG. But not likely because of #1.
3. One can wonder how such low docking energy for approved inhibitors is generated, and it is interesting to compare what the score would be from MD after g_mmpbsa; maybe something wrong was in the docking protocol.
4. It is interesting why the size of the box in Table 2 is so different for the same protein but just missense mutant forms?
5. Also, it is interesting to see conformations changes in spike structure according to interaction with inhibitors or molecular mechanism of inhibition and what is the difference with mutant forms given such short times run.

Is the work clearly and accurately presented and does it cite the current literature?

Yes

If applicable, is the statistical analysis and its interpretation appropriate?

Partly

Are all the source data underlying the results available to ensure full reproducibility?

No source data required

Is the study design appropriate and is the work technically sound?

Partly

Are the conclusions drawn adequately supported by the results?

No

Are sufficient details of methods and analysis provided to allow replication by others?

Yes

Reviewer Expertise:

NA

I confirm that I have read this submission and believe that I have an appropriate level of expertise to state that I do not consider it to be of an acceptable scientific standard, for reasons outlined above.

# Peer review: Reviewer response for version 1

**DOI:** 10.5256/f1000research.121103.r148621

**Published:** 2022-09-05

**Authors:** Nurdjannah Jane Niode

**Affiliations:** 1 Universitas Sam Ratulangi, Manado, Indonesia

This article explains about interactions of the surface glycoprotein of SARS-CoV-2 variants of concern (alpha, beta, delta, delta plus, gamma) with potential drugs for COVID-19 (remdesivir, baricitinib, favipiravir, lopinavir, dexamethasone) through structural modelling and molecular docking experiments.

 A. Is the work clearly and accurately presented and does it cite the current literature?

 Partly, because:

1. In Result and Discussion section, written that 'The reported mutations which had a frequency of occurrence of 50% and above were considered for surface glycoprotein modeling for VOCs (Table 1)'.
  Please explain further what the author means by frequency of occurrence of 50% and above, because the frequency is not mentioned in Table 1
2.  In MD Simulation and RSMD section written that 'MD simulation for favipiravir/Alpha variant was performed as it showed a significant binding affinity compared to other drugs under study.'
  Please explain the reason why the authors choose Favipiravir/Alpha variant among other variants for molecular dynamic simulation in the beginning of the MD simulation and RSMD paragraph.
3. Some references were taken from sources more than 10 years ago. If it's possible please replace the references with the latest references.

B. Are the conclusions drawn adequately supported by the results?

1. Partly, In conclusion section, its better to only present the results from the research (by removing the citation).
2. In the conclusion section in the abstract, please mention what drugs the authors mean by these drugs because there was no explanation in the beginning of the conclusion paragraph.

Is the work clearly and accurately presented and does it cite the current literature?

Partly

If applicable, is the statistical analysis and its interpretation appropriate?

Yes

Are all the source data underlying the results available to ensure full reproducibility?

Yes

Is the study design appropriate and is the work technically sound?

Yes

Are the conclusions drawn adequately supported by the results?

Partly

Are sufficient details of methods and analysis provided to allow replication by others?

Yes

Reviewer Expertise:

biology molecular (in infectious disease), dermatology and venereology, medical enthomology

I confirm that I have read this submission and believe that I have an appropriate level of expertise to confirm that it is of an acceptable scientific standard.

# Peer review: Reviewer response for version 1

**DOI:** 10.5256/f1000research.121103.r130114

**Published:** 2022-04-25

**Authors:** Akinwunmi Oluwaseun Adeoye

**Affiliations:** 1 Department of Biochemistry, Federal University Oye-Ekiti, Oye-Ekiti, Nigeria

This study describes the interaction of surface glycoprotein of SARS-CoV-2 variants of concern with potential drug candidates: a molecular docking study. The authors concluded that the mutations which occurred outside the receptor-binding domains of the surface glycoprotein of several variants of concern do not affect the binding affinity of these drugs. The study is well-designed and well written. The materials and methods section is clear and detailed. Information on multiple sequence alignment and structural analysis, as well as molecular dynamics simulation, is sufficient. The results presented are good as well as the discussion section. It is an interesting study that I believe falls within the scope of this journal. The study contains stimulating information for the readers of F1000Research with an interest in repurposing existing antiviral drugs with potential activity against SARS-CoV-2 variants of concern. This paper is suitable for publication in this journal.

 I have reviewed this article with keen interest. In general, the study design seems perfect. However, I observed in the structured abstract that the objective of the study was stated in the methods. From the study, the authors reported that the binding affinity of each drug for different variants was computed by assuming 100% binding affinity with SARS-CoV-2 wild type. Also, they considered the lowest binding energy and RMSD conformation as the most suitable docking pose. I believe that the drug molecule with the highest binding affinity to different variants of SARS-CoV-2 surface glycoproteins may not necessarily have the lowest binding energy and vice versa. The authors should have reported the binding affinities of the selected drugs with the SARS-CoV-2 surface glycoproteins separately in Kcal/mol from the docking study. Probably, the affinity of the drug molecules to the surface glycoprotein of SARS-CoV-2 variants of concern could be presented or represented in fold with respect to the SARS-CoV-2 wild type. The computation of the binding affinity of each drug concerning the assumption of 100% binding affinity with SARS-CoV-2 WT led to over 100% affinity of Baricitinib with beta variant, Favipiravir with alpha, beta, and delta variants as well as Remdesivir with the beta variant. What is the justification for these increases in affinity over 100% when compared with the reference SARS-CoV-2 wild type? Do any of the drugs alter the structures of SARS-CoV-2 surface glycoproteins?

Is the work clearly and accurately presented and does it cite the current literature?

Yes

If applicable, is the statistical analysis and its interpretation appropriate?

Yes

Are all the source data underlying the results available to ensure full reproducibility?

Yes

Is the study design appropriate and is the work technically sound?

Yes

Are the conclusions drawn adequately supported by the results?

Yes

Are sufficient details of methods and analysis provided to allow replication by others?

Yes

Reviewer Expertise:

Biomembrane, Molecular Toxicology and Computational Biology

I confirm that I have read this submission and believe that I have an appropriate level of expertise to confirm that it is of an acceptable scientific standard.
